# California and Oregon NICU Wildfire Disaster Preparedness Tools

**DOI:** 10.3390/children8060465

**Published:** 2021-06-01

**Authors:** Amy L. Ma, Mackenzie E. D. Loughland, Wannasiri Lapcharoensap, Dmitry Dukhovny, Henry C. Lee

**Affiliations:** 1 Department of Pediatrics, Stanford University, Stanford, CA 94305, USA; hclee@stanford.edu; 2 Department of Pediatrics, Division of Neonatology, Asante Rogue Regional Medical Center, Medford, OR 97504, USA; mackenzie.loughland@asante.org; 3 Department of Pediatrics, Oregon Health & Sciences University, Portland, OR 97239, USA; lapcharo@ohsu.edu (W.L.); dukhovny@ohsu.edu (D.D.); 4 Northwest Neonatal Improvement Priority Alliance, Portland, OR 97239, USA; 5 California Perinatal Quality Care Collaborative, Stanford, CA 94305, USA

**Keywords:** NICU, perinatal care, California, Oregon, Washington, wildfire, disaster preparedness

## Abstract

The 2020 wildfire season was devastating to the Western United States and affected the region’s NICUs. In this study, we ask the question, “what tools/strategies do medical professionals deem as important and most helpful as they are preparing for wildfire disaster response?” It is a follow up to our previous study: Learning from Wildfire Disaster Experience in California NICUs. We reevaluated how California NICUs dealt with the 2020 wildfires and expanded to Oregon and Southwest (SW) Washington NICUs. We conducted a survey with eleven Oregon and SW Washington NICUs about their wildfire evacuation preparedness. We also interviewed two neonatologists about their wildfire disaster experience evacuating their NICU or preparing to evacuate. Our findings suggest there is more work needed to fully prepare NICUs for wildfire disasters. We hope that by bringing light to the strategies used by affected clinicians, we can educate and support future NICU disaster preparedness responses.

## 1. Introduction

**Figure 1:**
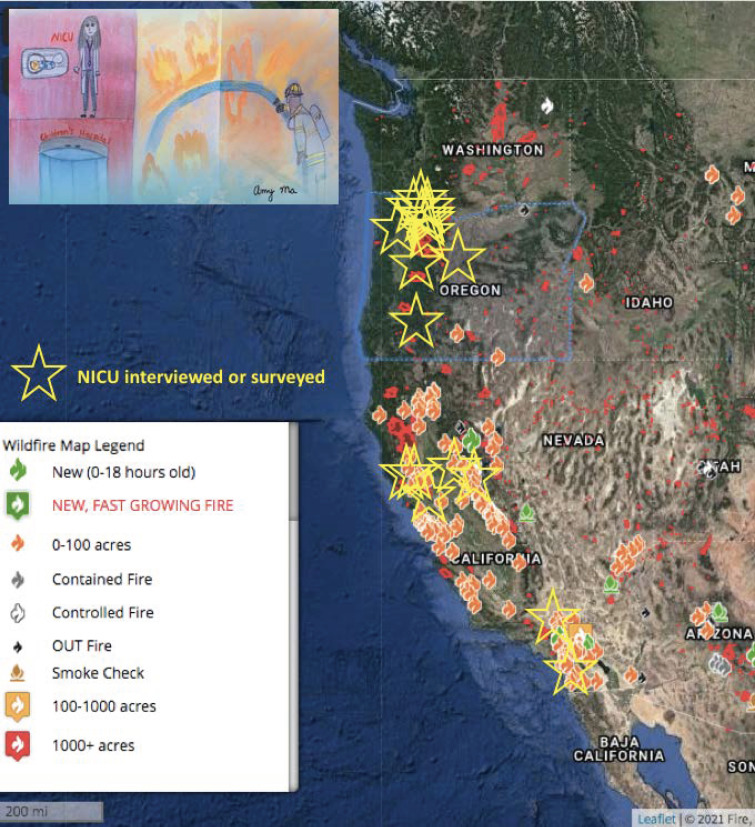
NICUs surveyed or interviewed in California, Oregon, and SW Washington. Fire map accessed in February 2021 [5].

Wildfires are natural disasters that greatly affect our world. Burning “out of control in a natural area, like a forest, grassland, or prairie,” they can be caused by humans’ improper campfire extinguishing [1]. Climate change, leading to warmer temperatures in spring and summer, decreased precipitation, extreme drought, high winds, and lightning strikes in the Western United States fuel wildfires [2,3,4]. Although it may seem like an unlikely threat, vulnerable patients in neonatal intensive care units (NICUs) are not exempt from being affected (Figure 1) [5].

California and the Pacific Northwest have experienced longer fire seasons each year; the timeline for the wildfire season had typically been four months but is now six to eight months [6]. Washington’s largest wildfire in recorded history occurred in 2014 and almost one million seven hundred thousand eighty-six acres burned in Oregon and Washington in 2015 [7]. The increased devastation and frequency of these natural disasters is alarming and novel, as is the impact of evacuation on the public. Medical professionals face the additional challenge of evacuating patients from hospitals to keep everyone safe.

The 2020 wildfire season was historically disastrous for Oregon, Colorado, and the West Coast in general, becoming the West Coast’s most active fire year on record [2,8]. By September 2020, twenty-one wildfires burned one million four thousand five hundred and sixty-two acres in Oregon, twice the ten-year average [9]. Forty thousand Oregonians evacuated their homes and 500,000 were placed under evacuation notice. By October, the year of 2020 had already brought “five of the six largest fires ever recorded in the state” of California [10]. 

Barnett notes how “hospital evacuation is given little thought, and minimal time is spent practicing even though healthcare organizations have identified a hazard vulnerability that may require a full-scale evacuation” [11]. Communication, preplanning, and leadership are contributing factors to successful evacuation, but these skills may not be practiced [11]. 

Disaster preparedness in NICUs does not only involve evacuation plans, but extends to redundant or back-up systems for power, medical gas, water, wall suction, and information technology. Fires both locally and distantly can lead to power outages, which may be sudden and also initiate an evacuation. Preparedness also manifests itself as live drills and the use of Tool Nomenclature to Categorize Infants and Evacuation Preparedness. The TRAIN™ tool (triage by resource allocation for in-patients) is a method used by clinicians to triage patients daily by assigning ambulance asset needs to prepare for potential evacuation [12]. Endorsed by the California Association of Neonatologists and the District IX AAP (American Academy of Pediatrics), the tool and modifications have been studied for neonatal and pediatric inpatients [12]. 

Improvements in disaster preparedness knowledge, skills, or attitude is invaluable to ensure optimal outcomes [13]. We performed a mixed methods study to examine how wildfire disaster preparedness was handled in the 2020 California, Oregon, and SW Washington wildfire disasters. Our goal is to inform future disaster preparedness efforts for NICU healthcare workers and administrators.

## 2. Materials and Methods

**Figure 2:**
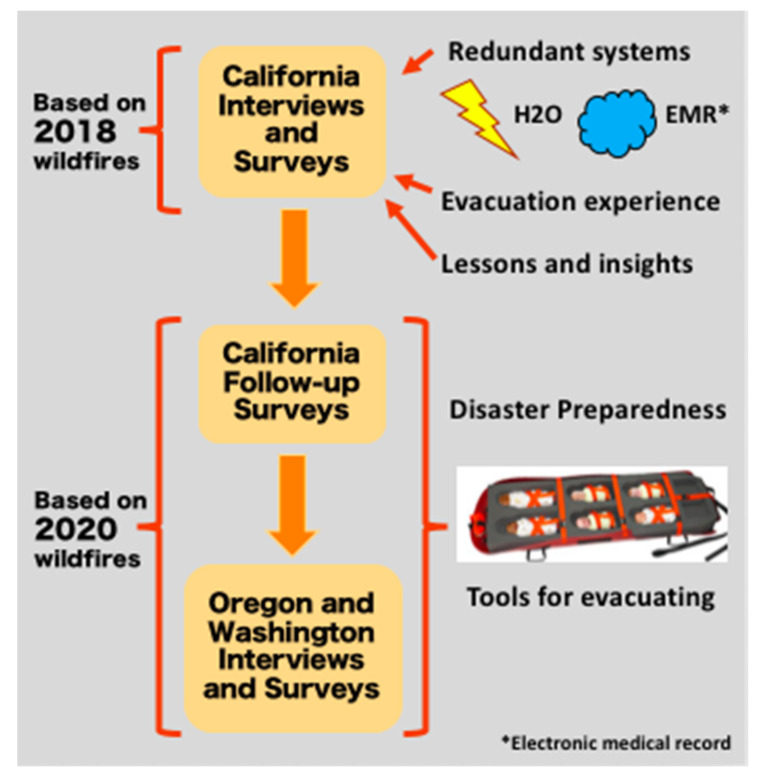
Methods schematic. The lightning bolt icon represents power; the cloud icon represents medical gas.

We designed a survey for the 2020 wildfire season based on the previous work on wildfire disaster experience in the California NICUs (Children 2020, 7(10), 155; doi:10.3390/children7100155). In addition to the California NICUs, the survey was distributed to members of the Northwest Neonatal Improvement Priority Alliance (NW IPA). NW IPA is an interdisciplinary collaboration of health care professionals and veteran NICU families. Comprised of 11 NICUs, it aims to improve the quality, safety, and outcomes for newborn infants in Oregon and Southwest Washington through education, research, quality improvement and advocacy. We also performed qualitative interviews with two Oregon neonatologists who shared their experience of the 2020 wildfire seasons: one of evacuating and one of preparing to evacuate. Data were collected from November 2020–January 2021. This study was conducted according to the guidelines of the Declaration of Helsinki and approved by the Stanford University Institutional Review Board (IRB protocol #32627; approval date 15 December 19). Interviewed or surveyed NICUs are depicted on the map above by a yellow star (Figure 1). By filling out the survey, participants consented to have the data entered studied. After filling out the survey, we emailed each participant a USD 25 gift card in appreciation for their time and effort. A summary of the workflow is pictured in Figure 2.

### 2.1. California Surveys

We surveyed neonatologists, nurse managers/directors, neonatal clinical nurse specialists, and NICU department managers online through Google Forms. Our sample pool was from participants that participated in past research regarding Learning from Wildfire Disaster Experience in California NICUs (Children 2020, 7(10), 155; doi:10.3390/children7100155). Four participants completed surveys from three institutions represented in our sample. Three institutions were a level III NICU and one of the three was a Level IV NICU as defined by the American Academy of Pediatrics (AAP). The AAP defines newborn levels of care as follows: Level I is for healthy well baby nursery care. Level II is specialty intensive care for sick and premature infants born at 32 weeks gestational age or greater and weighing 1500 g or more. Level III units provide comprehensive care for seriously ill newborns. Level IV is a regional NICU with the capabilities for major surgery, surgical repair of serious congenital heart anomalies, and a center with the capacity for extracorporeal membrane oxygenation (ECMO) [14].

Aside from demographic questions, the survey consisted of thirteen questions over three main sections: participant’s background, disaster preparedness and tools for evacuating a NICU due to a wildfire/preparing to evacuate (Appendix A: California Survey Questions). These questions were curated from results from the Learning from Wildfire Disaster Response in California NICUs study (Children 2020, 7(10), 155; doi:10.3390/children7100155).

Survey data were analyzed using qualitative research methods by being read iteratively to find common themes. We used a grounded theory approach based on our data. 

### 2.2. NW IPA Surveys

Consisting of fifteen questions, the survey was sent to the leads of the 11 NW IPA NICUs (Appendix B: NW IPA Survey Questions). We asked hospitals to fill out one per hospital as a team to ensure the most accurate responses from a multidisciplinary perspective. The survey was conducted online on Google Form. We received twelve responses to the NW IPA survey; one NICU submitted two responses, each conducted by a different individual. 

### 2.3. NW IPA Interviews

We interviewed two Oregon medical professionals, one about a NICU evacuation and another about preparing to evacuate. These interviews lasted an hour each and were audio-recorded through remote conferencing software. This was a semi-structured interview with six open-ended questions to facilitate discussion (Appendix C: NW IPA Interview Questions).

## 3. Results

### 3.1. California and NW IPA Surveys

Of the sixteen (four CA and twelve NW IPA) surveys completed, roles included NICU medical directors (*n* = 7), neonatologists (*n* = 7), nurse managers/directors (*n* = 6), neonatal clinical nurse specialists (*n* = 1), and NICU department managers (*n* = 1) over fourteen (three CA and eleven NW IPA) locations. 

### 3.2. CA and NW IPA Disaster Preparedness

#### 3.2.1. NICU Preparedness for a Wildfire Disaster

One of sixteen total responses said that their NICU is over prepared. Five NICUs are very prepared while five NICUs are somewhat prepared; four NICUs are a little prepared and one NICU is not prepared at all. 

#### 3.2.2. Tools Currently Available in NICUs for Evacuation Due to a Wildfire

California and NW IPA responses differed especially on TRAIN™ tool (triage by resource allocation for in-patients). All four California NICUs while none of the twelve NW IPA NICUs utilized the TRAIN™ tool. 

**Figure 3:**
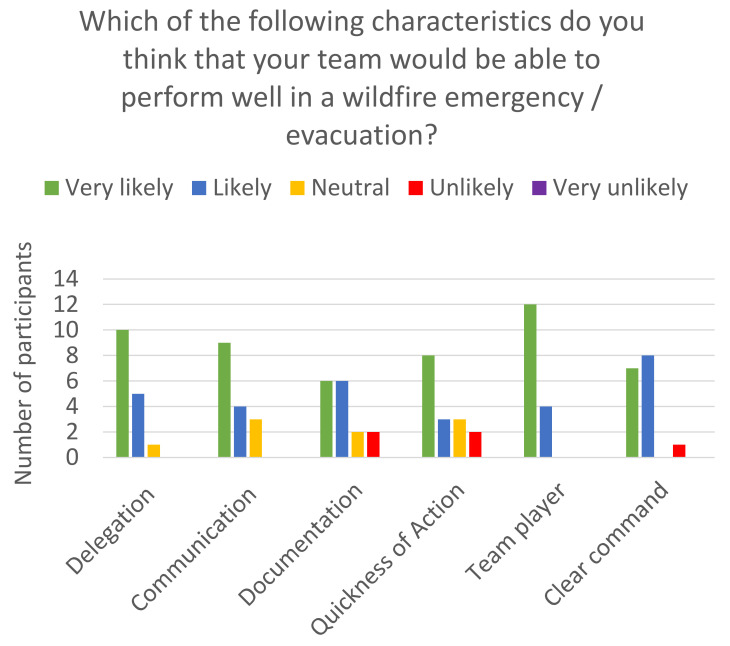
Projected team performance in wildfire emergency/evacuation among California, Oregon, and SW Washington NICUs. The majority of NICU medical professionals feel that their team is very likely to have good delegation, communication, quickness of action, being a team player during a wildfire emergency/evacuation.

Participants noted common tools and strategies, such as bassinets (*n* = 9), emergency backpacks (*n* = 5), Med Sleds^®^ (*n* = 4), baby aprons (*n* = 3), and the support and hard work of staff and fellow NICUs’ local collaboration (*n* = 2) (Figure 3). Other tools currently available in CA and NW IPA NICUs include the emergency staffing plan, emergency disaster plan that covers evacuation or a surge, supply lists, car seats, communication tree, shelter in place with air quality mitigation, transport teams, and Emergency Operating Command Center and Mission Control to help with capacity and bed management issues during times of strain and disaster. Another important “tool” that a participant said was available in their NICU was experience. A detailed evacuation plan was noted as currently available, as were evacuation tools/supplies/equipment. Essentials like travel/go bags of formula, blankets, diapers, and bottles were available in participants’ NICUs as well. The county office of emergency services was an additional tool noted as available in the NICU for evacuation due to a wildfire.

CA and NW IPA Tools for Evacuating a NICU due to a Wildfire/Preparing to Evacuate.

### 3.3. CA and NW IPA Evacuation Plans

#### 3.3.1. Plans to Transport the Babies Out of the Hospital

Five participants planned to use ambulances. Two planned to use a private car with car seats. Three answered ground/air/helipad transport. Six NICUs noted they would use one or both of the two specialized neonatal transport teams in the state. Two units planned to use Basic Life Support equipped ambulance teams. To transport the babies from the hospital to the evacuation vehicles, all sixteen have bassinets, four have Med-Sleds^®^, and three have aprons. Transport isolettes were also reported as available to two centers.

Five NICUs mentioned utilizing non-specific neonatal-pediatric critical care transport teams. One NICU is at a defend in place hospital which minimizes the need for evacuation, allowing patients to remain in place or relocate within the building [15]. Therefore, this NICU is unlikely to transfer babies. They would use advanced life support depending on the severity of patients. Two NICUs said they would use basic life support.

#### 3.3.2. How NICU Teams Identify Babies

All of the participant’s teams identify babies using identification (ID) bands. Three NICUs identify babies by placing stickers on the baby’s abdomen. One NICU triages based on acuity with colored spot on the ID band.

#### 3.3.3. Characteristics NICU Teams Would Be Able to Perform Well in a Wildfire Emergency/Evacuation

Figure 3, as seen above, shows participants beliefs about their team’s ability to perform well in a wildfire emergency/evacuation.

They are likely to have clear command. An equal number of participants believe that they are very likely or likely to have good documentation during a wildfire evacuation/emergency. Responses from CA follow up and NW IPA surveys (*N* = 16)

#### 3.3.4. Current Strategies or Equipment That Help NICUs Evacuate/Accept Babies Due to a Wildfire

Fifty percent of the participants said NICU transport—one specifying equipment (ground and air) with another specifying NICU transporters (limited number reserved for most critical). One participant said aprons, while another said evacuation trays, giraffe warmer with portable trolley attachment (limited number).

#### 3.3.5. Tools or Strategies That Medical Professionals Have Learned That They Would Like to Include in the Future

One participant spoke of implementing drills. Another said they now have pre-made downtime charting folders and have added H&P (History and Physical) and face sheets to their downtime computer to print in an emergency. The H&P gives patient history and pertinent patient information while the face sheet provides demographic and emergency contact information.

After surveying the NW IPA medical professionals, we wanted to gain anecdotal information about the wildfire disaster experience during the 2020 wildfires in Oregon. We interviewed a medical director whose NICU evacuated and a neonatologist whose NICU prepared for evacuation.

### 3.4. Oregon Interviews: Evacuation and Preparing for Evacuation

#### 3.4.1. Interview #1: Evacuating

The Story of Evacuation: A Level III NICU Medical Director’s collaborative experience planning the transport of high-risk neonates during an Oregon wildfire.

*This story is written in the perspective of the NICU medical director. Information was obtained from an interview conducted with medical director, Dr. Hillary Nicholson.

On the evening of Monday, 7 September 2020, Labor Day, wildfires burned in Oregon. By Wednesday, 9 September, our area hospital was in the Level 1 evacuation zone and told to be ready. Levels of evacuation are explained in Table 1.

Talking amongst hospital leadership about evacuation, staff designated each patient and their needs. All fourteen of our NICU patients were considered “red” because they could not be moved in a wheelchair.

Our nurses made bags of necessary supplies to bring with each baby and medical staff discussed if we should open a Level II NICU at Westside, a location far away from the fires, and have our staff go there to take care of the babies if space was limited at other NICUs. The Oregon Health Association authorized emergency approval to open an emergency level II NICU, but because of all the open bed spaces in the area, we elected not to pursue this option.

As the fire was encroaching and news that a wildfire was near our hospital, our medical staff weighed the prospect of evacuating to other area hospitals in Portland. Concerned about winds, we ultimately decided to transfer all the patients, evenly distributing them among different NICUs in the area based on where they lived. At that moment, forty NICU bed spaces were available throughout other NICUs in our region. Every family was very understanding, a big help. Three patients were nearing discharge, so we planned to transfer eleven patients that night and discharge the remaining three in the morning.

I worked with another neonatologist to arrange the transports that night, trying to avoid the hospital being under Level 2 evacuation where we would need to emergently transfer babies. Luckily, we did not have to Level 2 evacuate and our process was smoother and more cautious.

Still, the process was not easy. Due to lack of regional coordination, I had to call each receiving neonatologist independently saying, “I have this patient and need to evacuate them. Are you able to accept this patient?” Fortunately, because I knew everyone that we transferred babies to, the process went smoother than it could have.

Once we knew where each baby was going, we coordinated with specialized and skilled transport teams. The first baby left at 21:40 and a new transport team came approximately every hour to pick up another baby. The last baby was out at 4:10 in the morning. Eleven babies were transported. I was surprised at how quickly we were able to transport babies out.

Our staff stayed at our hospital; we relinquished all care to the receiving NICUs once transferred until the babies were brought back. There were no problems with the electronic medical record (EMR), another blessing. All area hospitals use EPIC and can see patient’s records through Care Everywhere.

Although the fires were partially contained and the wind changed directions, the air quality was still bad in the NICU and near the hospital. You could see smoke in the hospital, and it was hazy outside. Because of this, we were confident that we should not bring the babies back until later.

When the air quality improved, we started receiving patients back. A couple babies were not stable enough to bring back, so they stayed at the receiving hospital until they were clinically stable. The process of transferring the babies back was much longer; it took over a week. The babies nearing discharge went home from the receiving hospital.

##### Debriefing: What We Learned

The actual evacuation process went smoothly, but it would have been nice to keep the babies within our hospital organization system.

There are probably a lot of gaps I do not know about. We had the luxury of evacuating in a controlled fashion with time and calmness; we had more than twelve hours to get everyone out. If we had to evacuate in an emergency, we may have learned even more from an equipment and transport standpoint. Regular EMS transport teams, neonatologists, or bedside nurses would likely have transported the babies instead of the specialized transport teams. In terms of opening the Level II NICU, there likely would have been more hiccups. We still converse about potentially opening a Level II NICU in the future.

A list of things you need is important, as is having the right equipment and the ability to transport patients. Debriefing afterwards ensures you are always learning. Ask what went well and what you can improve upon.

#### 3.4.2. Interview #2: Preparing to Evacuate

The Story of Preparing for Evacuation: A Neonatologist’s experience during and after an Oregon wildfire.

*This story is written in the perspective of the neonatologist who was not in the unit but volunteered to help during the disaster response and conducted interviews and debriefings after the event. Information was obtained from an interview conducted with neonatologist, Dr. Mackenzie Loughland.

At 11 a.m. on Tuesday, 8 September 2020, our Trauma Program Manager received a call from her husband about the wildfires. During the hospital’s incident command meeting, she quickly stepped into the position of Emergency Preparedness Manager, a role that had been vacant for a couple of months. While I was not on call at the hospital the day of the wildfire, I was in town. After hearing there was a fire in a nearby town heading toward the hospital, I volunteered to assist. As next steps were being discussed among leadership, we waited for more information about whether we would need to evacuate or shelter in place. Code Triage Internal for Asante Ashland Community Hospital was called at 12:51 p.m., which quickly progressed to a system-wide incident command including Asante Rogue Regional Medical Center and Asante Three Rivers Medical Center. At 4:39 p.m., there was a call for providers who could be available.

While we waited, we planned for evacuation. Our hospital, located in southern Oregon, is remote from other surrounding NICUs. The closest NICU is 2.5 h away. We called individual NICUs to determine what beds were available. At this point there was no regional coordination for an event of this magnitude. An additional challenge was road closures due to the fires north and south causing us to be essentially cut off from the rest of the state, and air transport had been grounded due to smoke.

The clinical practice advisor, neonatologist and transport team nurse at the hospital developed an evacuation list with patient names, their respiratory support, equipment needs (i.e., isolettes vs. cribs), and the staff members who would transport those patients. We were lucky because we were well staffed. All day-shift staff were held over and our night-shift staff were able to make it to work. There were three neonatologists on service with one in-house and four others on standby, with cars and car seats ready to evacuate.

An organizational supply and triage system of pink, yellow and green was developed on the spot. Pink meant the baby was the most acute and needed the highest level of support. Yellow was intermediate and green was for feeder growers, babies without other medical concerns than learning to feed and increase weight. Overall, the acuity and census were relatively low in the unit that day. There were six pink babies, two yellow and eight green. Bedside nurses put together bags with necessary supplies for each of the sixteen neonates. Evacuation bags included a stethoscope, bulb suction, blood pressure cuff, thermometer, diapers, wipes, PPE, emergency code med sheet, formula, breast milk, feeding tube, IV pumps, supplies for ordered labs, daily progress note, face sheet with parent contact information and other supplies as needed.

We did not have a lot of time, so babies could not be transported one at a time. We organized eight teams with two patients per team. We considered the level of respiratory support, mode of transport (ambulance and transporters, bus or car and car seats), transport supplies (transport bags vs. off-unit bags), transport medication boxes and pulse oximetry to measure the oxygen level in the blood. The plan was for two staff members to go with the pink patients by ambulance. Six of the eight teams had the option to evacuate by private car or bus with a nurse and respiratory therapist or neonatologist in the vehicle and someone else driving.

In terms of transport from the NICU bed to the bus, car, or ambulance, the main NICU is on the ground floor, so the medical team could roll the more acute patients out the door. The second-floor babies would have been carried down the stairs since all were feeder growers without extensive support equipment.

As we were preparing to evacuate, leadership was evaluating the ability to evacuate a remote 300 bed hospital safely and successfully with only an hour’s notice. The roads were also congested with thousands of people evacuating their homes. The decision was made to shelter in place and that decision was communicated at 10 p.m. The fire department assured they could and would protect the hospital if we were in imminent danger. In the end, the winds shifted away from the hospital, the fire department contained the fire, and we did not have to evacuate.

##### Debriefing: Our Next Steps and What We Learned

After the incident, we debriefed and created a team to develop a NICU-specific evacuation policy and procedure for future incidences. We saved the supply list and evacuation plan created that day for future use until another tool is implemented. We attended a triage tool webinar and hope to implement a triage tool throughout our hospital system and throughout Oregon. A coordinated triage allows for quick and accurate communication between the NICU, hospital incident command and community services.

Education about HOSCAPs, Oregon’s hospital capacity web system, is also needed. NICUs in Oregon began using it at the beginning of the COVID-19 pandemic; however, it was not utilized that day. Quickly identifying available beds and hospitals impacted by other fires in the state is important, so NICUs can support each other and function in a coordinated way.

Communication flow was less efficient and disconnected because we did not have a clear communication structure or workflow within the NICU, and medical directors and physicians were not included in hospital incident command meetings. The communication silos from nursing leadership to shelter in place contradicted information the neonatologist received from medical staff leadership to evacuate. This was a major problem and a source of confusion in an already stressful and intense situation. The NICU needs an incident command communication workflow in addition to the hospital incident command. This will allow for more effective and efficient communication between hospital incident command and the NICU as we communicate the needs of the NICU. Additionally, having the NICU social worker in the unit, rather than in a different and unfamiliar unit, would have been helpful. Having a trusted and familiar person to communicate with the neonates’ families and assist staff in crisis is valuable.

Our NICU is at the beginning of its journey for disaster preparedness. This was not the first time our region has been threatened and it will not be the last. We will keep learning from our experiences. Before the next fire season, we hope to have a quick triage method and NICU-specific evacuation policy and procedure. A feasible state of readiness for an evacuation includes an EMR generating triage tool, the ability to triage by hand quickly on paper, and complete policies and procedures with supply lists, checklists and tools that are quick and easy to use in the moment. It is not feasible to have a bag packed for every patient for every moment for every disaster scenario but thinking through general scenarios helps create a framework. Then running simulations will help prepare us, so there is less thinking on our feet and more muscle memory with checklists and tools.

Additional reflections include the realization that knowledge is power against panic, so we need to keep everyone informed in a timely manner.

Being prepared at home is also important, and step number one for disaster preparedness. Evacuation plans for families and homes are critical, so we are ready to go to the hospital and be less worried about our families at home.

At a time when nearly 3000 people lost their homes to the wildfire, our NICU family pulled together and helped each other, and our vulnerable patients stay safe. Even though two NICU staff lost their homes, and many homes were threatened, nurses, RTs and doctors called in to see how they could help. As my colleague Dr. Sarah Kahn said after reflecting on the events, “We were literally willing to walk through fire for each other.”

## 4. Discussion

We appreciated the time taken by clinicians at two of the Oregon NICUs who had experienced recent critical circumstances during this past wildfire season. The interviews were insightful and detailed, and depicted two different cases and outcomes: one evacuation and one “shelter in place.” We also appreciate the California and NW IPA clinicians that filled out the survey.

This study brought to light that a problem with evacuation still exists; NICU specific evacuation plans are still not delineated in policies and procedures, as showcased by the interview stories. The themes expressed in both interviews were evacuation or preparing for evacuation, teamwork, preparedness and executing the plan to keep everyone safe. This phenomenon expands beyond Oregon and SW Washington. Medical professionals from University of Washington and Seattle Children’s Hospital wrote in their 2019 paper Identifying Crucial Equipment and Skills Needed to Evacuate Critically Ill Infants During Disasters: Using Nursing Expertise to Guide Training Targets, “There is limited evidence on what skills and bedside equipment are most important to include in disaster training and evacuation programs for critically ill infants” [17]. In their study, an expert panel created a checklist of skills for bedside registered nurses to evacuate a critically ill infant successfully. Across twenty-three surveys, participants rated the importance of the bedside equipment for evacuation and the challenges of intubating an unstable infant during a disaster. Like our study, Gray et al. found that documentation, communication, and decision making are important for success.

The findings apply to many geographic areas and different types of disasters/public health emergencies, not just the wildfires in the western United States. With crisis incidents occurring more frequently and having a great impact, advances in preparedness work extends beyond the United States. Investigators in Poland published a paper in 2020, Disaster Preparedness and Professional Competence among Healthcare Providers: Pilot Study Results recounting hospital preparedness and measuring healthcare workers competence [18]. Most respondents said that their workplace had a plan for dealing with mass-casualty incidents and disasters, but they had “not received training related to disaster preparedness” [18].

To give another global perspective, a 2020 study evaluated Saudi Arabia healthcare workers’ perceptions of preparedness and willingness to work during disasters in a survey; 62% of participants were willing to work unconditionally when facing natural disasters, showing how the medical community often sacrifices to help patients, even in times of crisis [19]. Evidence from our surveys and interviews showed Western United States NICUs healthcare workers also sacrificed their time and efforts to help. Even if they were not physically in the hospital or on call, they volunteered to help.

The perceptions of preparedness from the Saudi Arabia study did not have metrics of their NICUs being over prepared, very prepared, somewhat prepared, a little prepared, and not prepared at all. Still, the information about the healthcare workers’ willingness to work during disaster brings great hope. The medical community has teamwork, collaboration, and care for patients. We measured Western United States healthcare clinician’s perceptions of preparedness, highlighted in 3.1.1 NICU Preparedness for a wildfire disaster of our study. Even though 31.25% of NICUs are perceived to be very prepared, 31.25% are perceived to be somewhat prepared and another 25% are perceived to be a little prepared, work is being done to be more prepared through webinars, internal evaluation and debriefing, and education.

Colleagues working in this area have shared helpful materials on NICU disaster response and evacuation:

1. https://cchealth.org/ems/pdf/Pediatric-Neonatal-Disaster-References-Guide.pdf (accessed on 24 April 2021).
2. https://www.luriechildrens.org/globalassets/documents/emsc/disaster/other/nicuevacuationguidelines20093.pdf (accessed on 24 April 2021).

We hope that this paper can be a catalyst for communication between medical professionals and incident command leadership to make decisions on increasing their NICU disaster preparedness, including having live drills to practice evacuating their hospitals. Wildfires and natural disasters will continue to pose a threat, so it is vital to prepare and practice for the safety of the vulnerable neonatal patients as well as their families and medical staff.

## Figures and Tables

**Table 1:** Levels of evacuation [16].

Level 1	Level 2	Level 3
**Be Ready** for potential evacuation	**Be Set** to evacuate	**Go** evacuate now
Monitor emergency service information	Prepare to leave at a moment’s notice	Evacuate immediately
Prepare and conduct precautionary movement of patients	Significant danger to area	Danger is current or imminent
Notice via emergency notification system if conditions worsen	This may be the only notice you receive	Last notice you receive

## Data Availability

The data supporting these results are in possession of the authors. Interested parties can contact the corresponding author for further details including to request data for further research.

## Appendix A. California Survey Questions

### Appendix A.1. Participant’s Background

1. Please describe your role/occupation.
2. Which institution do you work at?
3. How many years have you been at this institution?
4. What level is your NICU?

### Appendix A.2. Disaster Preparedness

5. How prepared are you/your NICU for a wildfire disaster?
6. What comes to mind as the tools currently available in your NICU for evacuation due to a wildfire?

### Appendix A.3. Tools for Evacuating a NICU due to a Wildfire/Preparing to Evacuate

7. How do you plan to transport the babies out of the hospital?
8. How does your team identify babies?
9. Which of the following characteristics do you think that your team would be able to perform in a wildfire emergency/evacuation?
10. Does your NICU use: Bassinets, Med Sleds^®^, Backpacks (with formula, wipes, diapers, feeding tubes)?
11. Which of these tools and plans exist in your unit: TRAIN™ tool, Evacuation Plan—Horizontal (issues in NICU only), Evacuation Plan—Vertical (out of the building), Live Drills?
12. What are strategies or equipment that have not been mentioned do you wish you had to help your NICU evacuate/accept babies due to a wildfire?
13. Are there any tools or strategies that you have learned about that you would like to include in the future?

## Appendix B: NW IPA Survey Questions

1. Which NICU do you work at?
2. Name of who is filling out the survey
3. Please describe your role/occupation. Select all that apply.
  - NICU medical director
  - Neonatologist
  - Nurse manager/director
  - Neonatal clinical nurse specialist
  - NICU nurse
  - Patient care manager
  - NICU department manager
  - Other:
4. Contact email

### Appendix B.1. Disaster Preparedness

5. How prepared are you/your NICU for a wildfire disaster?
6. What comes to mind as the tools currently available in your NICU for evacuation due to a wildfire?

### Appendix B.2. Tools for Evacuating a NICU due to a Wildfire/Preparing to Evacuate

7. How do you plan to transport the babies out of the hospital?
8. How does your team identify babies?
  - ID bands
  - Place stickers on baby’s abdomen
  - Other:
9. Which of the following characteristics do you think that your team would be able to perform in a wildfire emergency/evacuation? Very likely; Likely; Neutral; Unlikely; Very unlikely
  - Delegation
  - Communication
  - Documentation
  - Quickness of action
  - Team player
  - Clear command structure
10. Does your NICU use: Bassinets, Med Sleds^®^, Backpacks (with formula, wipes, diapers, feeding tubes)? Check for yes. Leave blank for no.
11. Which of these tools and plans exist in your unit: TRAIN™ tool, Evacuation Plan—Horizontal (issues in NICU only), Evacuation Plan—Vertical (out of the building), Live Drills? Check for yes. Leave blank for no.
12. What are strategies or equipment that you already have that have not been mentioned that help your NICU evacuate/accept babies due to a wildfire?
13. What are strategies or equipment that have not been mentioned do you wish you had to help your NICU evacuate/accept babies due to a wildfire?
14. Are there any tools or strategies that you have learned about that you would like to include in the future?
15. Do you have any additional comments?

## Appendix C: NW IPA Interview Questions

### Appendix C.1. Participant’s Background

1. Please describe your role as it relates to the NICU evacuation that you will be describing.

### Appendix C.2. Evacuation Experience

2. Please describe your experience with the Fire Incident and the NICU Evacuation.

### Appendix C.3. Lessons and Insights

3. Can you describe any changes that your NICU implemented in your evacuation procedures after the experience with this incident?
4. Are there other aspects of disaster preparedness that you think are gaps in your NICU (Source: NICU Disaster Preparedness Survey—Gap Analysis)
5. Can you share any insights on how to better prepare for future disasters that can inform neonatal transport?
6. Is there anything else you would like to share?
